# *Barbell Technique* Associated With *Sinus Lift* for Horizontal and Vertical Guided Bone Regeneration—Case Report

**DOI:** 10.1155/crid/3253904

**Published:** 2025-05-22

**Authors:** Fernanda Calvo Costa, Thaís Cachuté Paradella, Fernando Vagner Raldi, Michelle Bianchi-de Moraes, Rodrigo Dias Nascimento

**Affiliations:** ^1^Department of Diagnosis and Surgery, Institute of Science and Technology, São Paulo State University (Unesp), São José dos Campos, São Paulo, Brazil; ^2^Department of Dental Materials and Prosthesis, Institute of Science and Technology, São Paulo State University (Unesp), São José dos Campos, São Paulo, Brazil

**Keywords:** alveolar bone grafting, alveolar bone loss, guided tissue regeneration

## Abstract

**Background:** Dental absences are a worldwide issue, affecting patients both socially and functionally. Implant rehabilitation is the gold standard treatment; however, prior bone reconstructions may be necessary. The Barbell Technique is a guided bone regeneration technique recommended for defects of various morphologies, allowing for vertical and horizontal unidirectional or bidirectional regeneration, that is, both on the buccal and palatal/lingual sides. The objective of this paper is to report a case of bone reconstruction in the posterior maxilla prior to implant rehabilitation, where the Barbell Technique was performed together with a sinus lift.

**Methods:** This study consists of a case report of a 77-year-old male patient with the absence of first and second right bicuspids and a history of implant losses in the area. The initial CT scan showed horizontal and vertical alveolar atrophy, making implant placement impossible. Regeneration was performed using a sinus lift for vertical recovery and the Barbell Technique for horizontal regeneration, using xenogeneic bone covered by a collagen membrane. After 6 months, a new CT scan suggested sufficient bone volume, and two implants were guidedly placed based on digital planning.

**Results:** Comparison of CT scans before and after the grafts showed a horizontal bone gain of 70% and a vertical gain of 33%. Six months after the reconstructive surgery, dental implants were placed, followed by the installation of individual crowns after 45 days. At the 24-month follow-up, no complications related to the implants or prostheses were observed.

**Conclusion:** In this clinical case, for the first time in the literature, the association of the techniques allowed the regeneration of the defect, with the subsequent placement of implants in the ideal position and prosthetic rehabilitation.

## 1. Introduction

Dental absences are a worldwide issue and lead to damage from the functional and aesthetic point of view. The success of the rehabilitation with implants depends, among other factors, on the technique employed during the moment of the installation of the implants, on the quality and amount of bone tissue available in the region to be rehabilitated [1, 2]. There are several techniques presented in the literature to correct bone defects of the alveolar bridge; among them are block grafts [3], guided bone regeneration (GBR) [4], *tenting pole technique* [5], *split bone block* [6], *sausage technique* [7], and alloplastic *scaffolds* [8]. Such techniques present limitations in the correction of horizontal defects unidirectionally, due to their difficulties in manipulating the palatal/lingual region. The problem is due to the fact that after the tooth extraction an alteration of the dimension of the alveolar bone occurs, specially due to the healing and bone remodelation [9], with the palatal bone loss being equivalent to 26% of the total, in the anterior region of the maxilla and, thus, it cannot be ignored [10]. From such a scenario, the *Barbell Technique* was idealized. Such a technique allows bidirectional reconstructions of the defects approaching simultaneously the lingual/palatal region. Another advantage of the technique is that it presents a device projected specially to support the membrane and the decompression of the region to be regenerated. It is a 1.5-mm diameter titanium screw with several lengths, having both local extremities to couple a capsule of poly-ether-ether-ketone (PEEK) which, in addition to preventing the compression of the tissues over the grafts, also promotes the protection of the soft tissue, avoiding damages to the surrounding soft tissue, as it occurs in other techniques [10]. Its versatility of application is another advantage. Recently, in addition to the possibility of appositional horizontal reconstructions in a uni or bidirectional form, its application for the treatment of vertical defects associated with horizontal defects has been described [11]. The posterior maxillary edentulous region is frequently prone to vertical bone deficiency due to the process of alveolar reabsorption associated with the pneumatization of the maxillary sinus [12]. However, the bone reconstructions performed in the interior of the maxillary sinus are well described in the literature with predictable and stable results. Therefore, this study reports for the first time in the literature a clinical case in which the technique was used in association with a standard maxillary sinus lift procedure, evaluating clinical and tomographic parameters with 24 months of follow-up.

## 2. Case Presentation

The present study was submitted to the Research Ethics Committee with Human Beings of the Dentistry School from São José dos Campos–UNESP, under the protocol CAAE: 78504824.9.0000.0077, and the patient signed the informed consent form.

**Figure 1:**
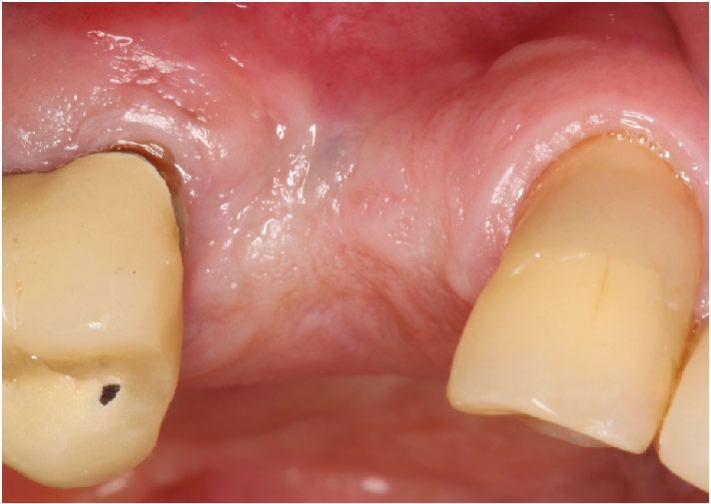
Initial clinical aspect, with the absence of the first and second right bicuspids and horizontal tissue deficiency.

**Figure 2:**
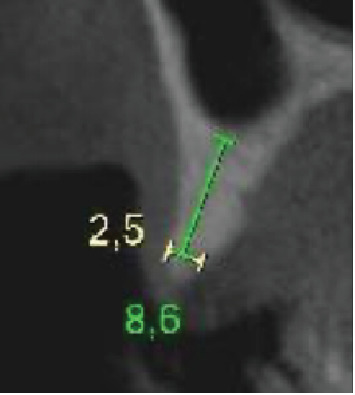
Initial CT of the region, highlighting measurements of 2.5 mm in thickness at the level of the crest and 8.6 mm in height.

The report is from a patient of the male gender, 77 years of age, who presented with tooth absences in the region of the first and second right bicuspids (Figure 1), with a history of implant losses and frustrated attempts at reconstruction in the region. The evaluation of the initial cone beam computed tomography scan highlighted horizontal and vertical alveolar atrophy in the region, which prevented the installation of implants in the ideal three-dimensional position, with measures of 2.5 and 8.6 mm, respectively (Figure 2), corresponding to the HAC 3 defect [13].

**Figure 3:**
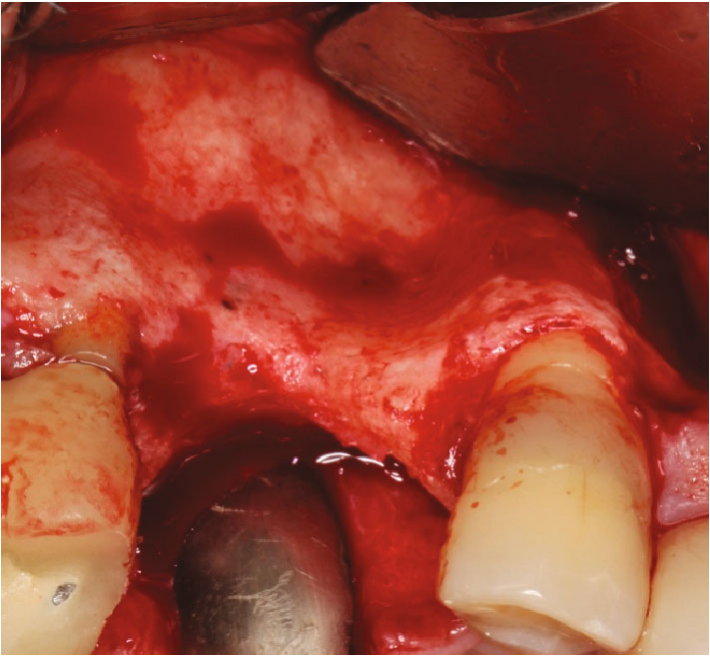
View of the horizontal deficiency after incision and mucoperiosteal flap elevation.

**Figure 4:**
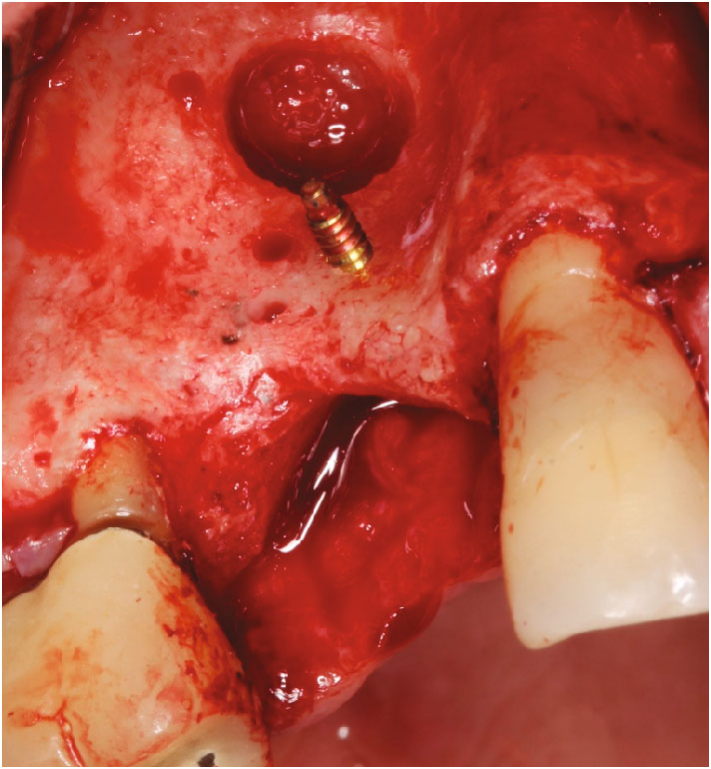
Window for sinus lift and an 8-mm barbell screw installed.

**Figure 5:**
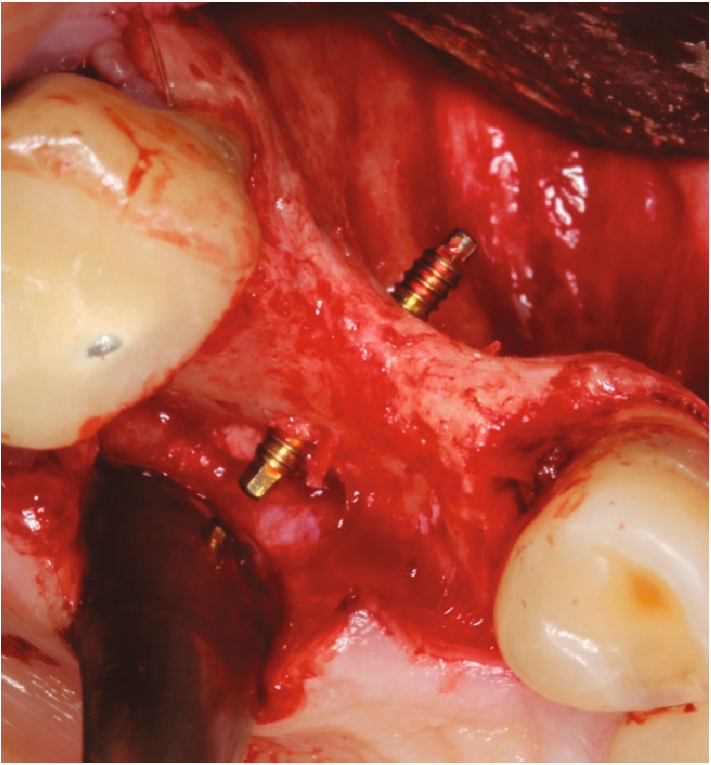
Occlusal view showing an 8-mm barbell screw installed transalveolarly.

**Figure 6:**
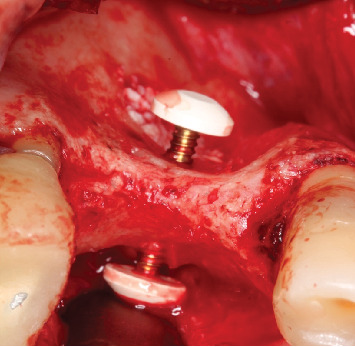
Occlusal view of the barbell screw and the PEEK capsules positioned.

**Figure 7:**
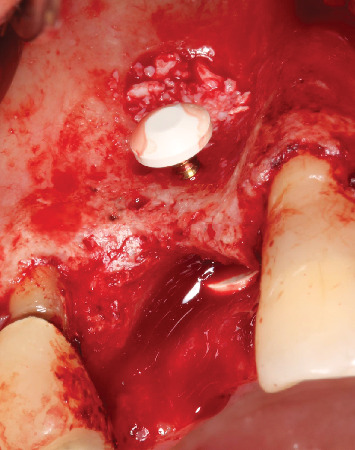
Sinus lift window filled with xenograft (Bio-Oss, Geistlich, Wolhusen, Switzerland) and barbell screw installed with PEEK capsules positioned.

**Figure 8:**
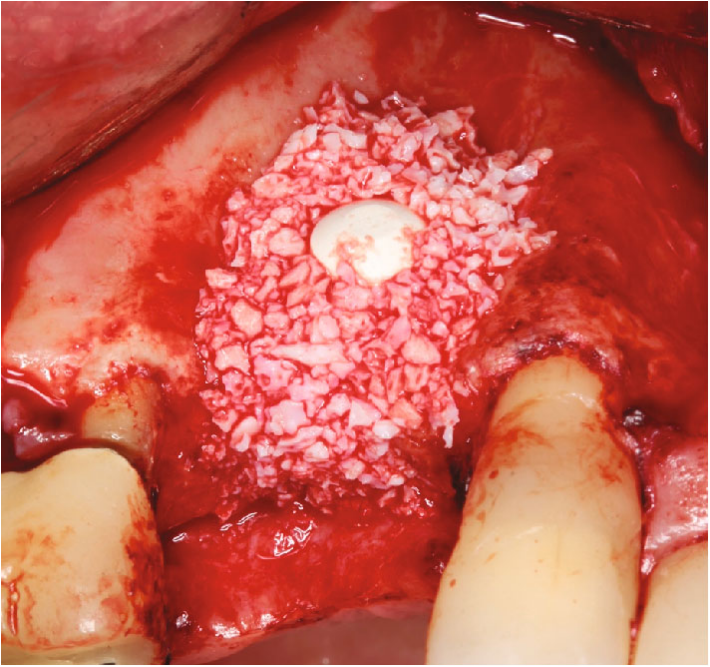
Frontal view after the buccal defect was filled with biomaterial (Bio-Oss, Geistlich, Wolhusen, Switzerland).

**Figure 9:**
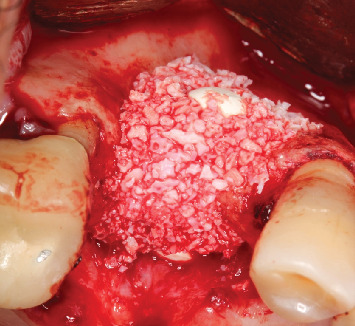
Occlusal view showing the positioning of the biomaterial (Bio-Oss, Geistlich, Wolhusen, Switzerland).

**Figure 10:**
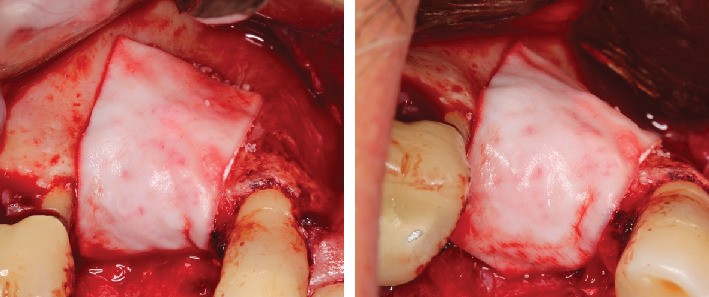
Coverage of the biomaterial with a natural porcine collagen membrane (Geistlich Bio-Gide, Wolhusen, Switzerland).

The procedure was performed under local anesthesia with 2% mepivacaine hydrochloride associated with epinephrine (1:100,000). A crestal incision was made with a 15c scalpel blade, along with a vertical relaxing incision on the mesial side and mucoperiosteal flap elevation (Figure 3). First, a bony window was opened on the buccal side using the Caldwell–Luc approach to perform a sinus lift, displacing the sinus membrane without perforating it. Next, decortication and perforation of the recipient bed were performed for the installation of the Barbell Technique screw horizontally in a transalveolar manner. An 8-mm screw was used (Figures 4 and 5), and after its placement, PEEK caps were inserted at both ends. The inorganic bone matrix (Bio-Oss, Geistlich, Wolhusen, Switzerland) was placed in the sinus lift cavity (Figures 6 and 7). Since it was an HAC 3 defect [13], the exclusive use of biomaterial was chosen, without the addition of an autogenous graft. Next, the bone graft was placed covering the buccal, crestal, and palatal regions of the alveolar ridge (Figures 8 and 9) and, finally, covered with a natural porcine collagen membrane (Geistlich Bio-Gide, Wolhusen, Switzerland) (Figure 10). No additional devices were used to fix the membrane, as the PEEK caps provided sufficient support and stability. After periosteal release, the flap was repositioned, sutures with 5.0 monofilament nylon thread were performed, and primary tension-free closure was achieved. As a postoperative guideline, the patient was instructed to take 500-mg amoxicillin every 8 h for 5 days, 100-mg nimesulide every 12 h for 3 days, and 500-mg paracetamol every 6 h in case of pain. The patient was also instructed to follow a soft diet and to perform mouth rinses with 0.12% chlorhexidine until the sutures were removed, which occurred after 15 days.

**Figure 11:**
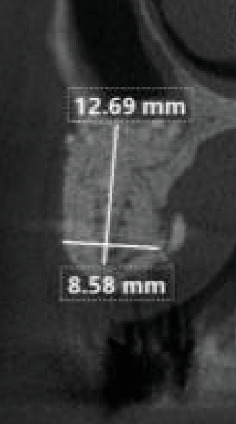
Postoperative tomography of the patient, with measurements of 8.5 mm horizontally and 12.6 mm vertically.

**Figure 12:**
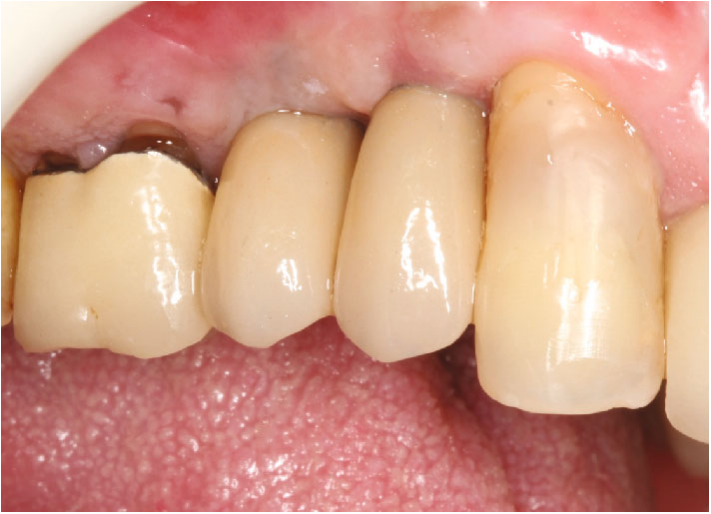
Frontal view after the installation of the zirconia single crowns.

**Figure 13:**
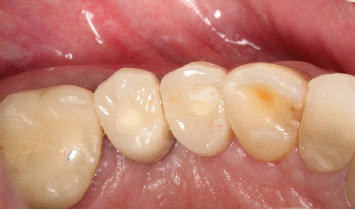
Occlusal view of the installed zirconia crowns.

**Figure 14:**
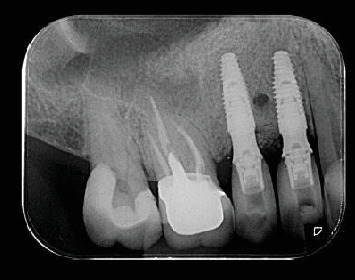
Periapical radiography of the implants placed with zirconia prosthesis rehabilitation.

After 6 months, a new tomographic examination showed sufficient bone volume with 8.58 mm of thickness at the crest level and 12.69 mm in the vertical direction (Figure 11). After prosthetic planning and the creation of a surgical guide, two Helix GM Acqua implants (Neodent, Curitiba, PR, Brazil) of 3.5 × 10 mm were installed in a prosthetically guided manner. The comparison of the tomographic scans before and after the grafts showed a horizontal bone gain of 6.0 mm (70%) and a vertical bone gain of 4 mm (33%). After 45 days, zirconia milled single crowns were installed (Figures 12, 13, and 14). Currently, the case is being followed for 24 months with peri-implant tissue maintenance and no complications related to the implant or prosthesis.

## 3. Discussion

Various techniques for vertical and horizontal bone augmentation are extensively documented in the literature. GBR is currently the most commonly used approach for appositional reconstructions. Once the principles of primary closure of the flap, promotion of angiogenesis, space maintenance, and wound stability are respected, the results are predictable, with low complication rates [14]. For space maintenance, the use of titanium-reinforced PTFE (polytetrafluoroethylene) membranes, both in dense and porous forms, is highlighted, as well as the use of absorbable natural or cross-linked collagen membranes supported by tent-type screws [15, 16].

Several studies have evaluated the results for horizontal gains using titanium-reinforced nonresorbable membranes combined with particulate xenograft grafts [7, 11, 15–18]. In one of these studies, average values of horizontal bone gain of 5.88 ± 1.17 mm were achieved, with no significant bone resorption reported in both clinical and radiographic aspects, no complications recorded, and all cases meeting survival criteria [17]. These results are similar to the findings in the present case, where a horizontal bone gain of 6.0 mm was achieved, corresponding to a 70% increase. However, it is worth noting that the gain obtained in the reported case occurred in a bidirectional manner, both buccal and palatal, in contrast to conventional horizontal bone reconstruction approaches that predominantly address the buccal aspect.

For this reason, the Barbell Technique has an important differential compared to other techniques, as the screw used to support the membrane can be applied transalveolarly, and its geometry allows the coupling of PEEK capsules on both the buccal and palatal sides, creating bilateral support for the collagen membrane and allowing a reconstructive approach from both sides [10]. Furthermore, it is important to emphasize that the use of titanium-reinforced membranes is associated with high rates of exposure, increasing the risk of infection, compromising healing, and negatively impacting the regenerated bone volume [11, 14, 16].

On the other hand, the use of natural porcine collagen membranes has shown significant reduction in exposures, and when they do occur, their rapid degradation decreases the risk of infection and allows soft tissue healing to occur effectively, without major complications [10, 11, 18, 19]. A limitation in the use of absorbable membranes in GBR techniques is the possibility of collapse due to compression of the grafted bone tissue, requiring the use of additional devices such as tent screws to maintain space and allow for nonpositioned regeneration [5, 20].

Such devices, like those used in titanium-reinforced membranes, tend to have a higher incidence of exposures and infections [11, 18, 19]. Therefore, the Barbell Technique, by using a PEEK device that offers greater biocompatibility with soft tissues by allowing the adhesion and proliferation of osteoblasts and fibroblasts on its surface, provides support to the absorbable membrane and prevents its collapse [21]. Thus, in addition to the use of natural collagen membranes favoring lower incidence of postoperative dehiscence and exposures, their structure for space maintenance in the Barbell Technique, due to the support and biocompatibility of the PEEK capsules, results in a solution with fewer complications, lower morbidity, and greater predictability of results.

In another study, a comparative analysis between GBR techniques using cross-linked membranes and natural collagen membranes was conducted, with the average horizontal gain obtained after the healing period being significantly lower in the group of patients undergoing GBR with cross-linked membranes (2.7 ± 1.8 mm, average gain of 83.2%) compared to patients treated with the sausage technique, using natural collagen membranes (5.3 ± 2.3 mm, average gain of 216.8%), corroborating the result found in the present case report [22]. In both GBR techniques evaluated (cross-linked membrane and natural collagen), a combination of autogenous and xenograft bone was used in a 1:1 ratio. In contrast, in the present case report, the decision was made to use only the xenograft biomaterial, due to the patient's ridge profile, classified as HAC 3, characterized by the presence of medullary bone between the buccal and palatal cortical bones [13].

The isolated use of osteoconductive biomaterial requires that the osteoinductive and osteogenic properties are derived from the recipient bed. In this way, the use of a collagen membrane with hydrophilic properties that allows revascularization through its surface, offering greater biocompatibility with soft tissue, has the ability to facilitate regeneration.

The horizontal bone gain observed in the present case is consistent with outcomes reported in previous studies employing the Barbell Technique, which demonstrated mean horizontal gains of 6.81 ± 1.33 [10] and 6.65 ± 1.09 mm [18]. In patients classified as HAC 3, the mean gain was 4.45 ± 0.75 mm [18]. In all cases, it was possible to install implants in appropriate positions, and there was no exposure of bone or membrane. All devices, including capsules and screws, were removed without complications for implant installation [10, 18].

The Barbell Technique also shows effective results for vertical reconstructions, where the presence of the vertical component reduces the regenerative potential of the defect, requiring the use of autogenous bone mixed with deproteinized bovine mineral matrix (DBMM) in a 50% proportion for each biomaterial [13]. After 9 months, the results showed significant improvement in aesthetics and bone volume, with the successful removal of the barbell devices and the installation of two new implants in satisfactory positions [11]. In the present clinical case, vertical bone deficiency was also present but in an inlay-type defect, which required a sinus lift. As it is a defect confined by bone walls, favoring the stability and vascularization of the graft material, the use of DBMM alone within the maxillary sinus, just like in the horizontal defect treated with the Barbell Technique, helped reduce the morbidity of the treatment, avoiding access to a second area for autogenous bone harvesting [13, 23, 24].

Although there is a clinical trial in which the Barbell Technique was employed for vertical ridge augmentation by using barbell screws placed within the maxillary sinus for bidirectional vertical gain [25], this is the first report in the literature describing the association of the Barbell Technique for horizontal augmentation with the conventional sinus lift technique.

Despite promising results, these need to be confirmed by other longitudinal studies, as it is a technique recently developed and described in the literature.

## 4. Conclusion

In the present clinical case, the use of the Barbell Technique combined with sinus lift facilitated the three-dimensional bone regeneration of the defect, allowing the installation of implants in the ideal position with subsequent prosthetic rehabilitation and stability of the result after 24 months.

## Data Availability

The data that support the findings of this study are available on request from the corresponding author. The data are not publicly available due to privacy or ethical restrictions.
